# Exploring the drivers of unmet need for contraception among adolescents and young women in Sierra Leone. a cross-sectional study

**DOI:** 10.1186/s40834-024-00286-8

**Published:** 2024-05-17

**Authors:** Augustus Osborne, Peter Bai James, Camilla Bangura, Jia B. Kangbai

**Affiliations:** 1 Department of Biological Sciences, School of Basic Sciences, Njala University, PMB, Freetown, Sierra Leone; 2 National Centre for Naturopathic Medicine, Faculty of Health, Southern Cross University, Lismore, Australia; 3 Faculty of Pharmaceutical Sciences, College of Medicine and Allied Health Sciences, University of Sierra Leone, Freetown, Sierra Leone; 4 Department of Environmental Health, School of Community Health Sciences, Njala University, PMB, Freetown, Sierra Leone

**Keywords:** Unmet, Contraception, Need, Adolescent, Young women, Sierra leone

## Abstract

**Background:**

Sierra Leone grapples with a concerning reality: a high unmet need for contraception among adolescents and young women (AYW). This translates to a multitude of unintended pregnancies, jeopardising their health, education, and overall life trajectory. To effectively address this challenge, we aim to examine the factors associated with the unmet need for contraception among AYW in Sierra Leone.

**Methods:**

The study analysed the 2019 Sierra Leone Demographic and Health Survey data. A total of 1,796 married and cohabiting AYW aged 15 to 24 years, representing the nationally representative sample, comprised the study. A multivariable binary regression analysis was used to explore the drivers of unmet needs for contraception. The regression results were presented using an adjusted odds ratio (AOR) with 95% confidence intervals (CI).

**Results:**

The study found that 29% of Sierra Leonean AYW had an unmet need for contraception. AYW with three or more births(AOR = 6.80, 95% CI = 3.97, 11.65), two births (AOR = 4.11, 95% CI = 2.50, 6.76), one birth (AOR = 4.40, 95% CI = 2.81, 6.88), heard family planning on TV last few months (AOR = 1.94, 95% CI = 0.98, 3.83), and are cohabiting (AOR = 1.88, 95% CI = 1.29, 2.75) had higher odds of unmet need for contraception. AYW who read the newspaper or magazine at least once a week (AOR = 0.11, 95% CI = 0.01, 1.10) had lower odds of unmet need for contraception.

**Conclusions:**

The study found a high unmet need among AYW in Sierra Leone, which indicates a significant gap between desired and actual contraceptive use, leading to unintended pregnancies and potentially adverse health and socio-economic consequences. Parity, media exposure and cohabitation were associated with a higher unmet need for contraception and newspaper/magazine readership was associated with a lower unmet need for contraception. The study highlights the need to increase access to affordable and diverse contraceptive options, especially in rural areas. Expand educational campaigns beyond TV to include print media and community-based interventions. Provide AYWs with knowledge and authority to make well-informed decisions around their sexual and reproductive well-being.

## Introduction

The World Health Organisation (WHO) defines women with an unmet need for contraception as those who are capable of reproduction and engage in sexual activity yet do not utilise any form of contraception and express a desire to either not have any more children or to postpone their subsequent pregnancy [1]. This indicator is crucial as it serves as the foundation for delivering contraceptive services and assessing a country’s adherence to the reproductive health rights of its population [2]. The widespread acceptance of contraceptives as an effective means of regulating fertility is widely recognized. It has a pivotal role in enhancing the health and overall welfare of women [3]. Furthermore, the significance of maternal health is emphasised in the Sustainable Development Goal (SDG) 3, which seeks to ensure the widespread availability of sexual and reproductive health services and reduce maternal death rates by 2030 [4, 5]. Unmet demands for contraception contribute to unsafe abortions, sexually transmitted infections, raised fertility rates associated with poverty, high maternal death rates, and poor employment [6].

Contraceptives are well recognised as crucial features for regulating fertility [7] and play a significant role in the reproductive health of women, particularly adolescent and young women (AYW) who wish to control the timing and number of children they have [2]. While contraceptives have been deemed successful in regulating fertility, their utilisation among AYW in SSA continues to be a pressing concern that requires immediate attention [8, 9]. Research has shown that AYW in SSA expresses a willingness to use contraceptives, but a significant number of them have difficulties accessing contraceptive services [10, 11]. An effective way to assess the difference between the demand for and availability of contraception is by calculating the unmet need for contraception [12].

Sierra Leone has a poor contraceptive prevalence rate and a significant unmet need for family planning compared to other countries in SSA [13]. Based on the 2019 Sierra Leone Demographic and Health Survey (SLDHS), a mere 21% of women aged 15–49 who were married or cohabiting were utilising any contraception, while 28% expressed a desire for family planning but were not receiving it [13]. These statistics suggest that there is an imbalance between the need for and availability of contraceptive services in the country, putting many women at risk of unwanted pregnancies and the resulting consequences.

Previous studies [12, 14–22] have found that the determinants of the unmet need for contraception are multifaceted and encompass various factors such as age, education, income level, marital status, employment, parity, awareness of family planning, exposure to media, place of residence (rural or urban), region, sex of household head, and respondent decision making.

Sierra Leone has made significant strides in recent years in promoting family planning and improving access to reproductive health services. Sierra Leone’s policy environment is generally supportive of family planning. The country has committed to the FP2030 initiative, which aims to ensure that all women and girls have access to modern contraception by 2030 [23]. Increasing the availability of contraceptives in health facilities, training health care providers on counselling and providing family planning services, community-based outreach programs to raise awareness about family planning, and educational campaigns to address misconceptions about contraception are the interventions being implemented to address the challenges of access, awareness, and knowledge of contraceptives in Sierra Leone [24]. Despite the progress that has been made, there are still a number of challenges to overcome in ensuring that all Sierra Leoneans have access to family planning services. Limited funding for family planning programs, weak health system infrastructure, cultural and religious barriers to family planning, and gender inequality are some of the barriers in Sierra Leone [24].

Despite the high unmet need, there is limited research on the specific factors that contribute to it in Sierra Leone. This makes it challenging to develop effective interventions to address the problem. Understanding the specific drivers of unmet needs can help to tailor interventions to address the most pressing issues. This study aims to explore the drivers associated with the unmet need for contraception among AYW in Sierra Leone, with the intention of providing suggestions to policymakers in the country. By shedding light on these complex and interconnected factors, we can pave the way for a future where young women in Sierra Leone have the power to make informed choices about their reproductive health and chart their destinies.

## Methods

### Data source and design

The 2019 SLDHS data was used for this study [25]. SLDHS was conducted over four months (from May 2019 to August 2019) to gather data on demographic, health, and nutritional factors among women, children, and men [13]. A cross-sectional design was adopted for the SLDHS, and respondents were sampled using a multistage sampling method. In the first stage, 578 enumeration areas (EAs), consisting of 214 urban and 364 rural regions, were selected. A systematic selection procedure was employed for the second stage to select 24 households from each EA. This selection process ultimately resulted in a sample size of 13,872 households [13]. The literature [13] provides a comprehensive explanation of the study methodology. This study included 1,796 married and cohabiting AYW aged 15 to 24 who had complete cases of variables of interest from the SLDHS. The dataset was accessed following the procedures outlined on the official DHS program website [25]. The study adhered to the Strengthening Reporting of Observational Studies in Epidemiology (STROBE) guidelines [26].

### Study variables

### Outcome variable

The study outcome variable was the unmet need for contraception. This was derived when AYW were asked whether they had an unmet need for contraception or not. The corresponding replies included never had sex, unmet need for spacing, unmet need for limiting, infecund/menopausal, no unmet need, using for limiting, using for spacing and not married and have not engaged in sexual intercourse in the past 30 days. Based on the classification of this variable in prior research [12, 14–18], AYW who had never engaged in sexual activity and those who were unable to conceive or experiencing menopause were excluded from the study. To get a binary outcome, the remaining responses were classified into two categories: 0 and 1. The value 0 reflected the absence of any unmet need, including the use of contraception for spacing or limiting purposes. On the other hand, value 1 indicated the presence of unmet need, either for spacing or limiting purposes.

### Explanatory variables

Seventeen explanatory variables were included in the study. The variables include the age of the women, place of residence, level of education, wealth index, employment status, region, parity, marital status, sex of household head, exposure to media (newspapers/magazines, radio, and television), exposure to family planning messages(radio, tv, newspapers/magazines and text messages) and decision making on respondent health care. These variables were selected based on their statistically significant association with the unmet need for contraception from previous studies [14–16, 19–22] and their availability in the DHS data. Table 1 shows the categories of the variables included in the study.

### Data analysis

The data was analysed using SPSS version 28. Percentages were used to present the prevalence of unmet need for contraception among AYW and their distribution across the explanatory variables. A chi-square test of independence was conducted to determine the variables significantly associated with the unmet need for contraception at *p* < 0.05. The variance inflation factor (VIF) was used to test for evidence of collinearity among the variables studied. The results showed that the highest and lowest VIF were 2.57 and 1.02. Hence, there was no evidence of high collinearity among the variables. Later, binary logistic regression analysis was performed to examine the variables associated with the unmet need for contraception. Two models were used. The first model was the bivariable analysis that examined the independent association between each explanatory variable and the unmet need for contraception. The last model was the multivariable analysis containing all the explanatory variables. The results were presented using adjusted odds ratio (AOR) with their respective 95% confidence interval (CI). Statistical significance was set at *p* < 0.05.

## Results

### Prevalence of unmet need for contraception among adolescents and young women in Sierra Leone

**Fig. 1:**
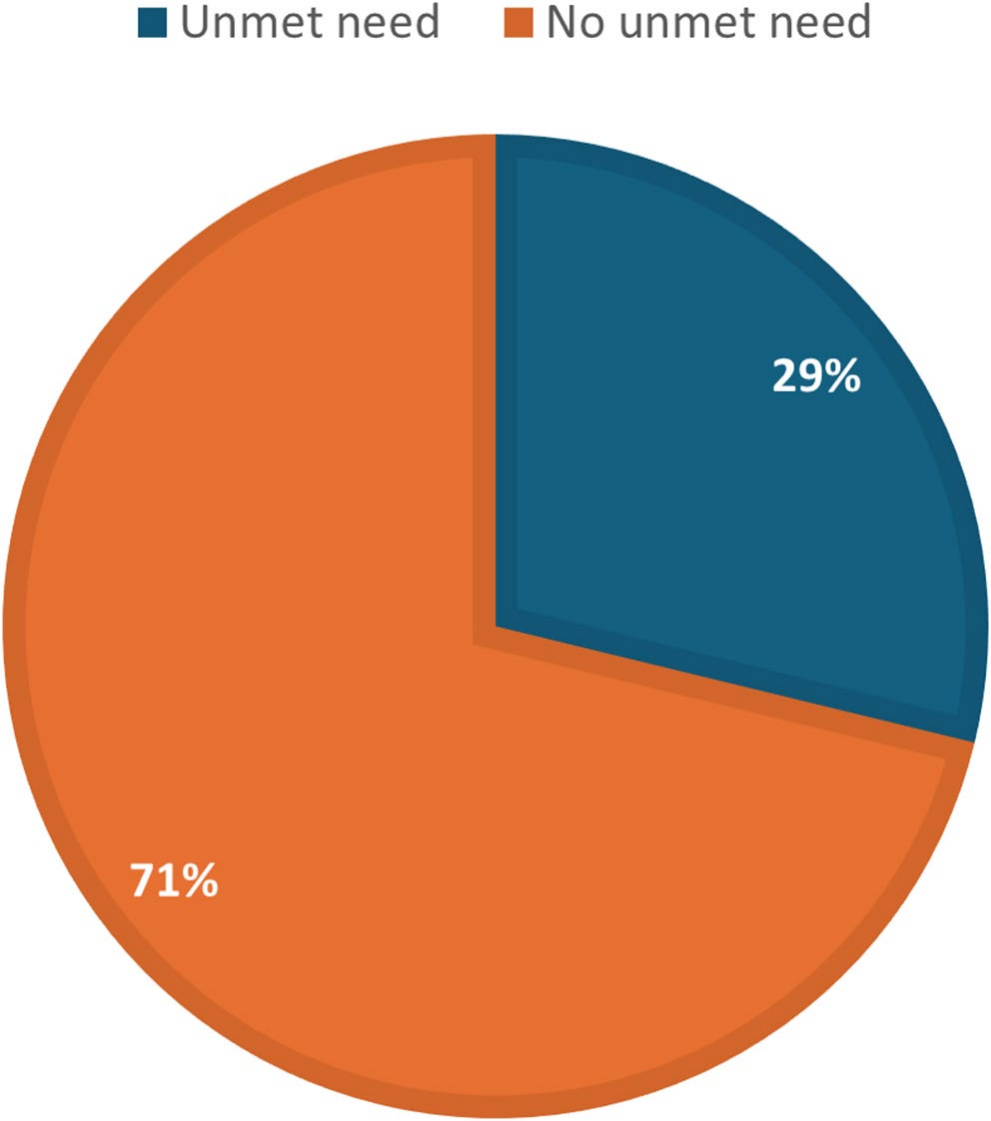
Prevalence of unmet need for contraception among adolescents and young women

Figure 1 shows the results of the prevalence of unmet need for contraception among adolescents and young women in Sierra Leone. The results indicated that 29% of Sierra Leonean AYW had an unmet need for contraception.

### Distribution of unmet need for contraception among adolescent and young women in Sierra Leone

Table 1 shows the distribution of the unmet need for contraception among AYW across the explanatory variables. AYW aged 20–24 (73.8%) live in rural areas (63,5%), have secondary/higher education(45.1%), and have males as household heads (80.3%) had high unmet need for contraception. There were variations in the unmet need for contraception in relation to exposure to family planning messages via TV, radio, newspaper or magazines, text messages and the frequency of reading newspapers or magazines, listening to radio, and watching television. Poorer AYW(23.8%), have one birth(46.8%), married (83.0%), currently working (67.5%), and respondent and others decide on respondents’ healthcare(94.2%) all had a high unmet need for contraception.

**Table 1:** Distribution of unmet need for contraception among adolescent and young women in Sierra Leone (*n* = 1796)

Variables	Category	Unmet need for contraception		*P*-value
		Yes *n*(%)	No *n*(%)	
**Age**				0.808
	15–19	135(26.2)	347(26.8)	
	20–24	383(73.8)	931(73.2)	
**Region**				0.653
	Eastern	90(18.4)	253(20.7)	
	Northern	115(19.1)	308(19.7)	
	Northwestern	133(25.0)	277(22.7)	
	Southern	102(17.1)	280(18.8)	
	Western	78(20.5)	160(18.2)	
**Place of residence**				0.705
	Urban	170(36.5)	401(35.3)	
	Rural	348(63.5)	877(64.7)	
**Level of education**				0.845
	No education	200(36.3)	507(37.7)	
	Primary	93(18.7)	234(19.0)	
	Secondary/higher	225(45.1)	537(43.4)	
**Sex of household head**				0.465
	Male	421(80.3)	1069(82.1)	
	Female	97(19.7)	209(17.9)	
**Frequency of reading newspaper or magazine**				0.031
	Not at all	488(94.1)	1231(95.7)	
	Less than once a week	29(5.8)	35(3.3)	
	At least once a week	1(0.1)	11(1.0)	
**Frequency of listening to radio**				0.830
	Not at all	329(62.2)	797(60.5)	
	Less than once a week	85(18.0)	226(19.1)	
	At least once a week	104(19.8)	225(20.4)	
**Frequency of watching television**				0.946
	Not at all	410(77.0)	1031(77.9)	
	Less than once a week	58(12.1)	133(11.6)	
	At least once a week	50(10.9)	114(10.5)	
**Wealth index**				0.996
	Poorest	114(20.0)	286(19.8)	
	Poorer	125(23.8)	318(24.2)	
	Middle	119(21.5)	273(21.4)	
	Richer	94(18.5)	239(19.2)	
	Richest	66(16.1)	162(15.5)	
**Parity**				< 0.001
	No birth	37(6.8)	274(20.8)	
	One birth	239(46.8)	494(38.6)	
	Two births	147(28.2)	353(28.4)	
	Three or more births	95(18.3)	157(12.3)	
**Heard family planning on the radio last few months**				0.124
	No	378(69.9)	987(74.5)	
	Yes	140(30.1)	291(25.5)	
**Heard family planning on TV last few months**				0.039
	No	480(90.3)	1222(93.9)	
	Yes	38(9.7)	56(6.1)	
**Heard family planning in a newspaper or magazine last few months**				0.443
	No	512(98.5)	1269(99.1)	
	Yes	6(1.5)	9(0.9)	
**Heard family planning through text messages over the last few months**				0.563
	No	507(97.4)	1250(97.9)	
	Yes	11(2.6)	28(2.1)	
**Marital status**				0.006
	Married	438(83.0)	1129(88.5)	
	Cohabiting	80(17.0)	149(11.5)	
**Current working status**				0.119
	No	170(32.5)	367(28.5)	
	Yes	348(67.5)	911(71.5)	
**Person who usually decides on the respondent’s health care**				0.513
	Respondent alone	33(5.8)	87(6.7)	
	Respondent and others	485(94.2)	1191(93.3)	

### Factors associated with unmet need for contraception among adolescents and young women in Sierra Leone

Table 2 shows the results of the factors associated with the unmet need for contraception among AYW in Sierra Leone. AYW with three or more births(AOR = 6.80, 95% CI = 3.97, 11.65), two births (AOR = 4.11, 95% CI = 2.50, 6.76), one birth (AOR = 4.40, 95% CI = 2.81, 6.88), heard family planning on TV last few months (AOR = 1.94, 95% CI = 0.98, 3.83), and are cohabiting (AOR = 1.88, 95% CI = 1.29, 2.75) had higher odds of unmet need for contraception. AYW who read the newspaper or magazine at least once a week (AOR = 0.11, 95% CI = 0.01, 1.10) had lower odds of unmet need for contraception.

**Table 2:** Factors associated with unmet need for contraception among adolescents and young women in Sierra Leone

Variables	Category	Unmet need for contraception	
		COR 95% CI	AOR 95% CI
**Age**			
	15–19	**Ref.**	**Ref.**
	20–24	1.03 (0.78, 1.35)	0.76 (0.56, 1.03)
**Region**			
	Eastern	**Ref.**	**Ref.**
	Northern	1.09 (0.73,1.61)	1.22 (0.80,1.88)
	Northwestern	1.23 (0.83,1.85)	1.44 (0.93,2.20)
	Southern	1.02 (0.68,1.51)	0.98 (0.64,1.51)
	Western	1.27 (0.79,2.03)	1.23 (0.68,2.21)
**Place of residence**			
	Urban	1.05 (0.80,1.37)	1.07 (0.68,1.71)
	Rural	**Ref.**	**Ref.**
**Level of education**			
	No education	**Ref.**	**Ref.**
	Primary	1.02 (0.73,1.41)	1.03 (0.72,1.46)
	Secondary/ higher	1.07 (0.82,1.40)	1.08 (0.80,1.46)
**Sex of household head**			
	Male	**Ref.**	**Ref.**
	Female	1.12 (0.82,1.53)	1.00 (0.73,1.37)
**Frequency of reading newspaper or magazine**			
	Not at all	**Ref.**	**Ref.**
	Less than once a week	1.77 (0.94,3.32)	2.04 (1.02,4.10)
	At least once a week	0.14** (0.01,1.19)	0.11** (0.01,1.10)
**Frequency of listening to radio**			
	Not at all	**Ref.**	**Ref.**
	Less than once a week	0.91 (0.67,1.24)	0.72 (0.48,1.09)
	At least once a week	0.94 (0.68,1.29)	0.81 (0.55,1.20)
**Frequency of watching television**			
	Not at all	**Ref.**	**Ref.**
	Less than once a week	1.05 (0.72,1.54)	0.84 (0.52,1.34)
	At least once a week	1.04 (0.70,1.54)	0.84 (0.47,1.50)
**Wealth index**			
	Poorest	0.97 (0.64,1.47)	0.95 (0.54,1.68)
	Poorer	0.94 (0.64,1.38)	1.27 (0.56,2.85)
	Middle	0.96 (0.63,1.46)	1.25 (0.59,2.66)
	Richer	0.92 (0.60,1.41)	1.20 (0.56,2.53)
	Richest	**Ref.**	**Ref.**
**Parity**			
	No birth	**Ref.**	**Ref.**
	One birth	3.72*** (2.41,5.76)	4.40*** (2.81,6.88)
	Two births	3.05 (1.89,4.91)	4.11** (2.50,6.76)
	Three or more births	4.55** (2.78,7.46)	6.80*** (3.97,11.65)
**Heard family planning on the radio last few months**			
	No	**Ref.**	**Ref.**
	Yes	1.25 (0.93,1.68)	1.10 (0.77,1.56)
**Heard family planning on TV last few months**			
	No	**Ref.**	**Ref.**
	Yes	1.66* (1.02,2.72)	1.94* (0.98,3.83)
**Heard family planning in a newspaper or magazine last few months**			
	No	**Ref.**	**Ref.**
	Yes	1.58 (0.48,5.24)	1.77 (0.59,5.33)
**Heard family planning through text messages over the last few months**			
	No	**Ref.**	**Ref.**
	Yes	1.25 (0.57,2.74)	1.02 (0.40,2.64)
**Marital status**			
	Married	**Ref.**	**Ref.**
	Cohabiting	1.57*** (1.13,2.18)	1.88*** (1.29,2.75)
**Current working status**			
	No	**Ref.**	**Ref.**
	Yes	0.82 (0.64,1.05)	0.78 (0.60,1.02)
**Person who usually decides on the respondent’s health care**			
	Respondent alone	0.85 (0.54,1.36)	0.82 (0.50,1.33)
	Respondent and others	**Ref.**	**Ref.**

aOR: adjusted odds ratio; cOR: crude odds ratio; 95% CI: 95% Confidence Interval; ref: reference category; * *p* < 0.05, ** p<; 0.01, *** p<; 0.001

## Discussion

The study examined the unmet need for contraception among married and cohabiting AYW in Sierra Leone. Our study found a high (29%) prevalence of unmet need for contraception among AYWs in Sierra Leone. The findings of our study are higher than the overall prevalence of unmet need for contraception in high fertility countries in SSA [15], Ethiopia [22], and Nigeria [27]. The findings from our study, however, are lower than the findings reported in Papua New Guinea [14], Burundi [28], and Ghana [21]. Several reasons contribute to the high unmet need for contraception among AYWs in Sierra Leone. Many AYWs, especially in rural areas, may lack access to health facilities offering family planning services. Long distances and transportation costs can be significant hurdles [29]. Many AYWs lack accurate information about their bodies, reproductive health, and contraceptive options due to limited access to quality CSE in schools and communities [30]. Misconceptions and negative attitudes towards contraception, fueled by cultural beliefs and social norms, can deter AYWs from seeking or using them [30]. Even when available, contraceptives may be unaffordable for many AYWs, especially those from low-income families [31]. These factors interact and reinforce each other, creating a complex landscape that fuels the high unmet need for contraception among AYWs in Sierra Leone. Addressing this issue requires a multifaceted approach that tackles access, knowledge, socio-economic barriers, and policy gaps, working with communities, healthcare systems, and policymakers to create an environment where AYWs can make informed choices about their sexual and reproductive health.

The study found that AYW with child/children had higher odds of unmet need for contraception than those with no births. The findings from our study are consistent with the previous studies [14, 15]. Raising multiple children can strain finances, making it challenging to afford contraceptives, especially if they are not subsidised or readily available. Cultural norms or power dynamics within relationships might restrict their ability to negotiate condom use or access other methods independently [32]. Fear of judgment or repercussions from family or community members for using contraception might deter them from seeking or using methods [33]. Some healthcare providers might hold negative attitudes towards young mothers seeking contraception, leading to inadequate counselling or limited method options offered [34]. Previous negative experiences with specific methods (e.g., side effects) might make them hesitant to try contraception again [34]. Addressing this issue requires interventions that combine increased access to affordable methods, comprehensive sexuality education, and empowering AYWs to make informed choices about their health and fertility.

The study found that AYW who are cohabiting had higher odds of unmet need for contraception than those who are married. The findings from our study are consistent with the previous studies [14, 15]. Unmarried AYWs might face objections from their partners in using contraceptives [35]. Young women in cohabiting relationships might have less financial autonomy compared to married women, making it harder to afford contraceptives independently [36]. Unmarried young women might have less family support compared to married women, reducing their access to financial resources or encouragement to prioritise their reproductive health. Addressing this issue requires ensuring equal access to services, as well as addressing socio-cultural stigma and power dynamics. Empowering these young women with information, negotiation skills, and access to confidential healthcare services is crucial in meeting their reproductive health needs.

Our study found that AYWs who heard family planning messages on TV had higher odds of unmet need for contraception than those who didn’t. While it might seem counterintuitive, It’s important to remember that correlation doesn’t necessarily imply causation [37], meaning exposure to information on TV doesn’t automatically translate to access or actual use. Hearing about family planning on TV might raise awareness and desire for contraception, but it doesn’t guarantee access to services or the specific methods they need. Our study also found that AYWs who read newspapers or magazines at least once a week have lower odds of unmet need for contraception than those who don’t. Newspapers and magazines may offer diverse viewpoints on reproductive health and contraception, challenging myths and stigma and promoting informed decision-making [38]. Exposure to accurate information can build confidence in understanding individual needs and navigating healthcare systems to access desired methods [39]. Regular newspaper or magazine readership generally suggests higher education levels and potentially better incomes, leading to greater opportunity and ability to afford contraception. Future research should explore specific mechanisms at play and consider how to improve access to accurate information and empower all AYWs, regardless of their media consumption habits, to make informed choices about their sexual and reproductive health.

### Policy and practice implications

Based on the study findings, several policy and practice implications can be considered to address the 29% unmet need for contraception among adolescents and young women in Sierra Leone. Government and partner organisations should dedicate resources to expand services, improve supply chains, and subsidise contraceptives for those who cannot afford them. Integrate family planning into national health priorities and budgets. Promote gender equality and empower AYWs through CSE that includes information about contraceptive methods and empowers them to negotiate their sexual health needs. Engage community leaders and religious groups to address misconceptions and stigma around contraception. Expand family planning services in health facilities, schools, and community settings, including mobile clinics and outreach programs. Train healthcare providers on AYWs’ specific needs and ensure respectful and non-judgmental counselling. They should offer a wide range of contraceptive methods by ensuring that all approved methods are available, including long-acting reversible contraceptives (LARCs) and emergency contraception—train providers in counselling and fitting different methods to address individual needs and preferences. Integrate CSE into school curricula and community programs. Equip AYWs with knowledge about their bodies, reproductive health, and negotiating power within relationships. Foster peer support networks and safe spaces for AYWs to discuss their concerns and access information. By addressing these policy and practice recommendations, Sierra Leone can move towards reducing the unmet need for contraception and ensuring access to sexual and reproductive health services for all AYWs, ultimately contributing to their well-being and improved life outcomes.

### Strengths and limitations

The key strength of this study is the use of the SLDHS, which provides information on a large and representative sample of AYWs across Sierra Leone, allowing for generalizable conclusions about the population. The survey collects a wide range of demographic, socio-economic, and reproductive health data, enabling the exploration of various factors associated with the unmet need for contraception. The study, however, has some limitations. The survey cannot establish causal relationships between factors and unmet needs. More research using longitudinal or intervention studies is needed. Reliance on self-reported data might introduce biases due to recall errors or social desirability. The survey may not capture specific reasons for unmet needs beyond broad categories like media exposure or cohabitation. Qualitative research may provide deeper insights. This study offers a valuable starting point for exploring the drivers of the unmet need for contraception among AYWs in Sierra Leone. However, its limitations highlight the need for further research using complementary methods and more recent data to gain a comprehensive understanding of the issue and develop effective interventions.

## Conclusion

The study found a high unmet need among AYW in Sierra Leone, which indicates a significant gap between desired and actual contraceptive use, leading to unintended pregnancies and potentially adverse health and socio-economic consequences. Parity, media exposure and cohabitation were associated with a higher unmet need for contraception and newspaper/magazine readership was associated with a lower unmet need for contraception. The study highlights the need to increase access to affordable and diverse contraceptive options, especially in rural areas. Expand educational campaigns beyond TV to include print media and community-based interventions. Empower AYWs with information and agency to make informed choices about their sexual and reproductive health.

## Data Availability

Data is publicly available via the measure dhs website at https://dhspr ogram.com/data/available-datasets.cfm.

## Abbreviations

AOR: Adjusted Odds Ratio
AYW: Adolescent and Young Women
CI: Confidence Interval
COR: Crude Odds Ratio
CSE: Comprehensive Sexual Education
EA: Enumeration areas
SLDHS: Sierra Leone Demographic and Health Survey
SSA: Sub-Saharan Africa

## References

[1] Indicator Metadata Registry Details [Internet]. [cited 2024 Feb 11]. https://www.who.int/data/gho/indicator-metadata-registry/imr-details/3414.

[2] Juarez F, Gayet C, Mejia-Pailles G (2018). Factors associated with unmet need for contraception in Mexico: evidence from the National Survey of Demographic Dynamics 2014. BMC Public Health.

[3] Ahmed S, Li Q, Liu L, Tsui AO (2012). Maternal deaths averted by contraceptive use: an analysis of 172 countries. Lancet.

[4] Health in 2015. from MDGs to SDGs [Internet]. [cited 2024 Feb 11]. https://www.who.int/data/gho/publications/mdgs-sdgs.

[5] Grove J, Claeson M, Bryce J, Amouzou A, Boerma T, Waiswa P (2015). Maternal, newborn, and child health and the Sustainable Development Goals—a call for sustained and improved measurement. Lancet.

[6] AS25[12June. 2012].pdf [Internet]. [cited 2024 Feb 11]. https://dhsprogram.com/pubs/pdf/AS25/AS25%5B12June2012%5D.pdf.

[7] Darroch JE (2013). Trends in contraceptive use. Contraception.

[8] Behrman JA, Wright KQ, Grant MJ, Soler-Hampejsek E (2018). Trends in Modern Contraceptive Use among Young Adult women in sub-saharan Africa 1990 to 2014. Stud Fam Plann.

[9] Radovich E, Dennis ML, Wong KLM, Ali M, Lynch CA, Cleland J (2018). Who meets the contraceptive needs of Young women in Sub-saharan Africa?. J Adolesc Health.

[10] Durowade KA, Omokanye LO, Elegbede OE, Adetokunbo S, Olomofe CO, Ajiboye AD (2017). Barriers to contraceptive uptake among women of reproductive age in a semi-urban community of Ekiti State, Southwest Nigeria. Ethiop J Health Sci.

[11] Makola L, Mlangeni L, Mabaso M, Chibi B, Sokhela Z, Silimfe Z (2019). Predictors of contraceptive use among adolescent girls and young women (AGYW) aged 15 to 24 years in South Africa: results from the 2012 national population-based household survey. BMC Womens Health.

[12] Machiyama K, Casterline JB, Mumah JN, Huda FA, Obare F, Odwe G (2017). Reasons for unmet need for family planning, with attention to the measurement of fertility preferences: protocol for a multi-site cohort study. Reproductive Health.

[13] Sierra Leone Demographic. and Health Survey 2019 [FR365].

[14] Agyekum AK, Adde KS, Aboagye RG, Salihu T, Seidu AA, Ahinkorah BO (2022). Unmet need for contraception and its associated factors among women in Papua New Guinea: analysis from the demographic and health survey. Reproductive Health.

[15] Ahinkorah BO (2020). Predictors of unmet need for contraception among adolescent girls and young women in selected high fertility countries in sub-saharan Africa: a multilevel mixed effects analysis. PLoS ONE.

[16] Ahinkorah BO, Ameyaw EK, Seidu AA (2020). Socio-economic and demographic predictors of unmet need for contraception among young women in sub-saharan Africa: evidence from cross-sectional surveys. Reproductive Health.

[17] Yalew M, Adane B, Kefale B, Damtie Y (2020). Individual and community-level factors associated with unmet need for contraception among reproductive-age women in Ethiopia; a multilevel analysis of 2016 Ethiopia demographic and Health Survey. BMC Public Health.

[18] Yaya S, Ghose B (2018). Prevalence of unmet need for contraception and its association with unwanted pregnancy among married women in Angola. PLoS ONE.

[19] Dingeta T, Oljira L, Worku A, Berhane Y (2019). Unmet need for Contraception among Young Married women in Eastern Ethiopia. OAJC.

[20] Sidze EM, Lardoux S, Speizer IS, Faye CM, Mutua MM, Badji F (2014). Young women’s Access to and use of contraceptives: the role of Providers’ restrictions in Urban Senegal. IPSRH.

[21] Wulifan JK, Mazalale J, Kambala C, Angko W, Asante J, Kpinpuo S (2019). Prevalence and determinants of unmet need for family planning among married women in Ghana-a multinomial logistic regression analysis of the GDHS, 2014. Contracept Reproductive Med.

[22] Tadele A, Abebaw D, Ali R (2019). Predictors of unmet need for family planning among all women of reproductive age in Ethiopia. Contracept Reproductive Med.

[23] Family Planning. and the 2030 Agenda for Sustainable Development (Data Booklet) [Internet]. United Nations; 2019 [cited 2024 Feb 19]. https://www.un-ilibrary.org/content/books/9789210045124.

[24] UNFPA Sierra Leone [Internet]. 2017 [cited 2024 Feb 23]. Family Planning. https://sierraleone.unfpa.org/en/topics/family-planning-6.

[25] The DHS. Program - Quality information to plan, monitor and improve population, health, and nutrition programs [Internet]. [cited 2024 Jan 9]. https://www.dhsprogram.com/.

[26] Von Elm E, Altman DG, Egger M, Pocock SJ, Gøtzsche PC, Vandenbroucke JP (2008). The strengthening the reporting of Observational studies in Epidemiology (STROBE) statement: guidelines for reporting observational studies. J Clin Epidemiol.

[27] Solanke BL, Oyinlola FF, Oyeleye OJ, Ilesanmi BB (2019). Maternal and community factors associated with unmet contraceptive need among childbearing women in Northern Nigeria. Contracept Reproductive Med.

[28] Nzokirishaka A, Itua I (2018). Determinants of unmet need for family planning among married women of reproductive age in Burundi: a cross-sectional study. Contracept Reproductive Med.

[29] Sserwanja Q, Musaba MW, Mutisya LM, Mukunya D (2022). Rural-urban correlates of modern contraceptives utilization among adolescents in Zambia: a national cross-sectional survey. BMC Womens Health.

[30] Massaquoi H, Atuhaire C, Chinkonono GS, Christensen BN, Bradby H, Cumber SN (2021). Exploring health-seeking behavior among adolescent mothers during the Ebola epidemic in western rural district of Freetown, Sierra Leone. BMC Pregnancy Childbirth.

[31] Ngacha JK, Ayah R (2022). Assessing the cost-effectiveness of contraceptive methods from a health provider perspective: case study of Kiambu County Hospital, Kenya. Reproductive Health.

[32] Solanke BL, Kupoluyi JA, Awoleye AF, Adewole OE, Babalola OB (2023). Women’s ability to negotiate safer sex with partners by contraceptive status among a nationally representative sample of married women in Nigeria. Contracept Reproductive Med.

[33] Wondimagegne YA, Debelew GT, Koricha ZB (2023). Barriers to contraceptive use among secondary school adolescents in Gedeo Zone, South Ethiopia: a formative qualitative study. BMJ Open.

[34] Gele AA, Shrestha M, Sheikh NS, Qureshi SA (2022). Pregnant and powerless: exploring barriers to contraceptive use among women in Mogadishu, Somalia. Health Serv Res Managerial Epidemiol.

[35] Hassan EE, Ghazawy ER, Amein NM (2017). Currently married women with an Unmet need for Contraception in Minia Governorate, Egypt: Profile and determinants. Open J Prev Med.

[36] Bogale B, Wondafrash M, Tilahun T, Girma E (2011). Married women’s decision-making power on modern contraceptive use in urban and rural southern Ethiopia. BMC Public Health.

[37] Correlation vs Causation [Internet]. [cited 2024 Feb 11]. https://www.jmp.com/en_au/statistics-knowledge-portal/what-is-correlation/correlation-vs-causation.html.

[38] Ahmed M, Seid A (2020). Association between exposure to Mass Media Family planning messages and Utilization of Modern Contraceptive among Urban and Rural Youth women in Ethiopia. Int J Womens Health.

[39] Sserwanja Q, Turimumahoro P, Nuwabaine L, Kamara K, Musaba MW (2022). Association between exposure to family planning messages on different mass media channels and the utilization of modern contraceptives among young women in Sierra Leone: insights from the 2019 Sierra Leone Demographic Health Survey. BMC Womens Health.

